# Utilizing principal component analysis in the identification of clinically relevant changes in patient HLA single antigen bead solid phase testing patterns

**DOI:** 10.1371/journal.pone.0288743

**Published:** 2023-10-26

**Authors:** Caleb Cornaby, Eric T. Weimer

**Affiliations:** 1 Histocompatibility & Diagnostic Immunology Laboratory, Children’s Hospital of Los Angeles, Los Angeles, California, United States of America; 2 Department of Pathology and Laboratory Medicine, University of Southern California Keck School of Medicine, Los Angeles, California, United States of America; 3 Molecular Immunology Laboratory, McLendon Clinical Laboratories, UNC Health, Chapel Hill, North Carolina, United States of America; 4 Department of Pathology & Laboratory Medicine, the University of North Carolina at Chapel Hill School of Medicine, Chapel Hill, North Carolina, United States of America; Stellenbosch University, SOUTH AFRICA

## Abstract

**Background:**

HLA antibody testing is essential for successful solid-organ allocation, patient monitoring post-transplant, and risk assessment for both solid-organ and hematopoietic transplant patients. Luminex solid-phase testing is the most common method for identifying HLA antibody specificities, making it one of the most complex immunoassays as each panel contains over 90 specificities for both HLA class I and HLA class II with most of the analysis being performed manually in the vendor-provided software. Principal component analysis (PCA), used in machine learning, is a feature extraction method often utilized to assess data with many variables.

**Methods & findings:**

In our study, solid organ transplant patients who exhibited HLA donor-specific antibodies (DSAs) were used to characterize the utility of PCA-derived analysis when compared to a control group of post-transplant and pre-transplant patients. ROC analysis was utilized to determine a potential threshold for the PCA-derived analysis that would indicate a significant change in a patient’s single antigen bead pattern. To evaluate if the algorithm could identify differences in patterns on HLA class I and HLA class II single antigen bead results using the optimized threshold, HLA antibody test results were analyzed using PCA-derived analysis and compared to the clinical results for each patient sample. The PCA-derived algorithm had a sensitivity of 100% (95% CI, 73.54%-100%), a specificity of 75% (95% CI, 56.30%-92.54%), with a PPV of 65% (95% CI, 52.50%-83.90%) and an NPV of 100%, in identifying new reactivity that differed from the patients historic HLA antibody pattern. Additionally, PCA-derived analysis was utilized to assess the potential over-reactivity of single antigen beads for both HLA class I and HLA class II antibody panels. This assessment of antibody results identified several beads in both the HLA class I and HLA class II antibody panel which exhibit over reactivity from 2018 to the present time.

**Conclusions:**

PCA-derived analysis would be ideal to help automatically identify patient samples that have an HLA antibody pattern of reactivity consistent with their history and those which exhibit changes in their antibody patterns which could include donor-specific antibodies, *de novo* HLA antibodies, and assay interference. A similar method could also be applied to evaluate the over-reactivity of beads in the HLA solid phase assays which would be beneficial for lot comparisons and instructive for transplant centers to better understand which beads are more prone to exhibiting over-reactivity and impact patient care.

## Introduction

HLA antibody testing first became recognized as clinically relevant in transplant medicine in the 1960s [1] and since then studies for all transplanted solid-organs have found that monitoring for anti-HLA antibodies is important for patient clinical care and prognosis [2, 3]. Clinical testing most widely used for detecting HLA antibodies are solid phase multiplex-based assays. Serum test results using these assays influence virtual crossmatch estimations, identification of unacceptable antigens, and characterization of donor-specific antibodies [4, 5].

Machine learning algorithms often use principal component analysis (PCA) and resultant matrices while processing input data sets. This is done as data sets analyzed by machine learning algorithms are often extremely large and cumbersome. PCA, as a data reduction technique, assists with extracting the most influential variables of a given data set. PCA is used in a wide variety of applications including image analysis software, such as facial recognition, finance evaluation, data exploration, and cyber security risk assessment [6–8].

Luminex-based solid-phase HLA antibody testing is a complex test performed in the clinical histocompatibility laboratory. Both HLA class I and HLA class II antibody assays can detect over 96 antibody specificities each with more bead specificities that are becoming available and employed in patient testing. When comparing patient historic antibody test results to current results, many medical laboratory scientists utilize the recommended company-provided software packages to visually examine samples and determine clinical significance based on their transplant center and laboratory standards of practice. Our study evaluated the application of a dimensionality reduction technique, PCA, to approach data analysis as a proof of concept. The goal was to determine if PCA-assisted data analysis could help in identifying clinically relevant changes in patient samples. Additionally, our analysis explores the use of PCA in identifying potentially over-reactive single antigen beads from a large transplant center data cohort.

## Materials and methods

### Samples

Patient samples were selected from solid organ pre-transplant, solid organ post-transplant, and hematopoietic stem cell transplant patients. All patient samples were collected and tested between January 2018 and March 2022 at McLendon Laboratories, UNC Hospital, Chapel Hill, NC. Patient data and sample results were collected per the UNC internal review board (IRB)-approved study parameters (IRB# 16–3116). The samples included in the proof-of-principle runs using PCA with our established thresholds were deidentified after all data had been compiled and analyzed. This was done because the clinical results needed to be extracted and paired with the PCA analysis for predicting clinically impactful changes in HLA antibodies. All other samples were deidentified after data collection and before PCA analysis.

### Solid phase testing

Solid phase testing for HLA antibodies was performed using HLA single antigen bead class I and class II assays (One Lambda) on the Flexmap 3D platform (Luminex). Sample testing was performed using patient serum and tested as recommended by the assay manufacturer [9–11].

### Bioinformatics

Machine learning algorithms often utilize matrices of covariance to identify differences between occurrences, images, or patterns that have multiple variables, in our study we utilized a similar approach to investigate MFI values from HLA solid phase single antigen bead results. Patient results for solid phase single antigen bead testing and sample identifiers were obtained using the Fusion software (v4.2, OneLambda). MFI values were obtained by exporting all test samples for the given time to.csv files. These.csv files were then concatenated utilizing R. The pre-transplant control subjects, post-transplant control subjects, and patients with donor-specific antibodies (DSA) were all de-identified and assigned a study number. All samples were assigned a sample study number per IRB protocol. Patient sample results were compiled, de-identified, and quality control samples removed using R (version 4.1.1). Analysis and grading of sample results based on the Euclidean distance was performed blinded from the review and clinical resulting process.

### Statistical analysis

Principal component analysis was performed using GraphPad Prism (version 9.4.1) and R (version 4.1.1). Euclidean distance analysis based on principal component values was performed using the equation, $Euclidean\;distance\;(ED)=\sqrt{{\left(X_{1}-X_{2}\right)}^{2}+{\left(Y_{1}-Y_{2}\right)}^{2}}$, as has previously been established and described for the analysis of principal component-derived values [12, 13]. The distance ratio of samples was calculated as the Euclidean distance of the sample of interest divided by the average Euclidean distance of the other patient samples. ROC analysis was performed using GraphPad Prism (version 9.4.1). The paired t-test or Mann-Whitney U test was performed when appropriate. For all statistical tests, calculated P-values < 0.05 were considered significant.

## Results

Machine learning algorithms utilize matrices of covariance to identify differences between occurrences, images, or patterns that have multiple variables. This is often referred to as the principal component analysis (PCA) method which utilizes the variance across multiple variables of multiple training samples to show similarity or dissimilarity between compared patterns from future samples [14]. In the PCA method, the Eigenvectors are calculated from the covariance matrix and then the unique features are displayed on a lower dimensional plane, typically with each plane, referred to as a principal component, accounting for a percentage of the variance observed across the samples compared [14, 15]. One way to determine if the pattern is similar or dissimilar is to measure the distance on the resultant planes used between the reference samples and the sample in question. The distance is referred to as the Euclidean distance if this is calculated on a two-dimensional plane [6]. Our study was designed to evaluate the utility of PCA in identifying clinically significant differences in solid-phase HLA antibody testing results using this method.

**Fig 1:**
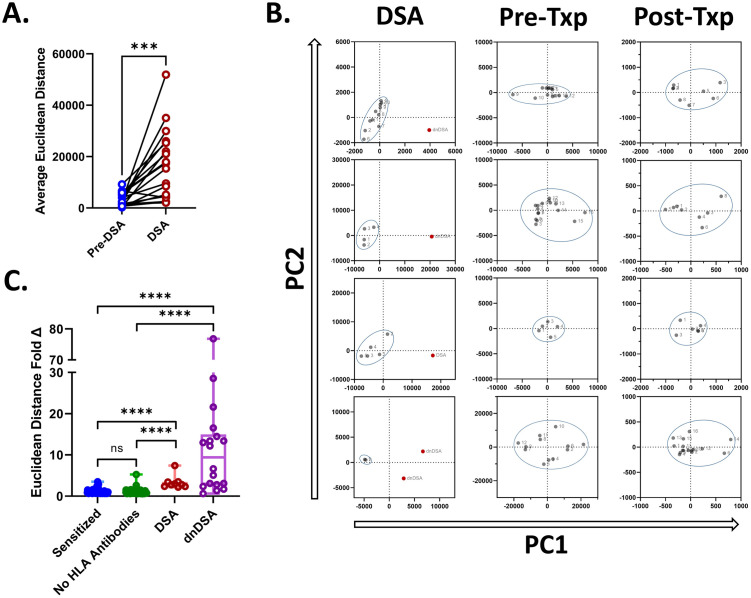
Principal component analysis of HLA class I results demonstrate that samples with DSA have higher Euclidean distances. (A) For patients with class I DSAs, the average Euclidean distance observed was higher in DSA samples compared to pre-DSA samples. (B) Representative PCA dot plots for PC1 and PC2 are displayed for DSA, highly sensitized pre-transplant and low reactive post-transplant sample cases. (C) The fold difference observed between the average Euclidean distance of each sample type and the overall average Euclidean distance between patient samples was compared. ***p-value < 0.005, ****p-value <0.0001.

To initially evaluate if this method could be appropriately applied to HLA antibody results, 25 patients that were positive for HLA donor-specific antibodies (DSA) between January 2019 and December 2021 were selected. PCA analysis was performed on each patient’s historic pre-DSA samples and the sample in which DSA was identified. Samples with DSA present a difference in pattern compared to the patient’s previous historic samples and are clinically significant. The average Euclidean distance was calculated post-PCA analysis between each of the historic samples. This was then compared to the average Euclidean distance exhibited between the DSA sample and each of the historic samples. This was performed for HLA class I (Fig 1A) DSAs. We found that the Euclidean distance was significantly higher for the DSA samples when they were compared to the historic pre-DSA sample Euclidean distances for our patient cohort for HLA class I (paired t-test, p < 0.0005). To further evaluate if this method could distinguish this known reactivity difference compared to samples that displayed a similar antibody pattern, two control cohorts of patient samples were also compared. Sensitized pre-transplant patients, comprising 159 patient samples from 13 different patients, were utilized as one control group. The second control group was composed of post-transplant patient samples that lacked reactivity, totaling 163 patient samples among 20 different patients.

PCA analysis was performed to compare samples for each patient in both cohorts. Then the Euclidean distance was calculated for HLA class I samples. Examples of how the PCA analysis visually appears for select patients for the DSA cohort and the two control cohorts are displayed for HLA class I (Fig 1B). To determine if there was a significant difference between sample Euclidean distances post PCA analysis among the cohort with DSA and the two control cohorts, the fold difference between each sample and the average Euclidean distance observed for each patient was calculated for HLA class I (Fig 1C). Our cohort of patients with DSAs was further sub-grouped by whether the DSA identified was historical *de novo* DSA (dnDSA) or was DSA that was present before the patient’s transplant.

**Fig 2:**
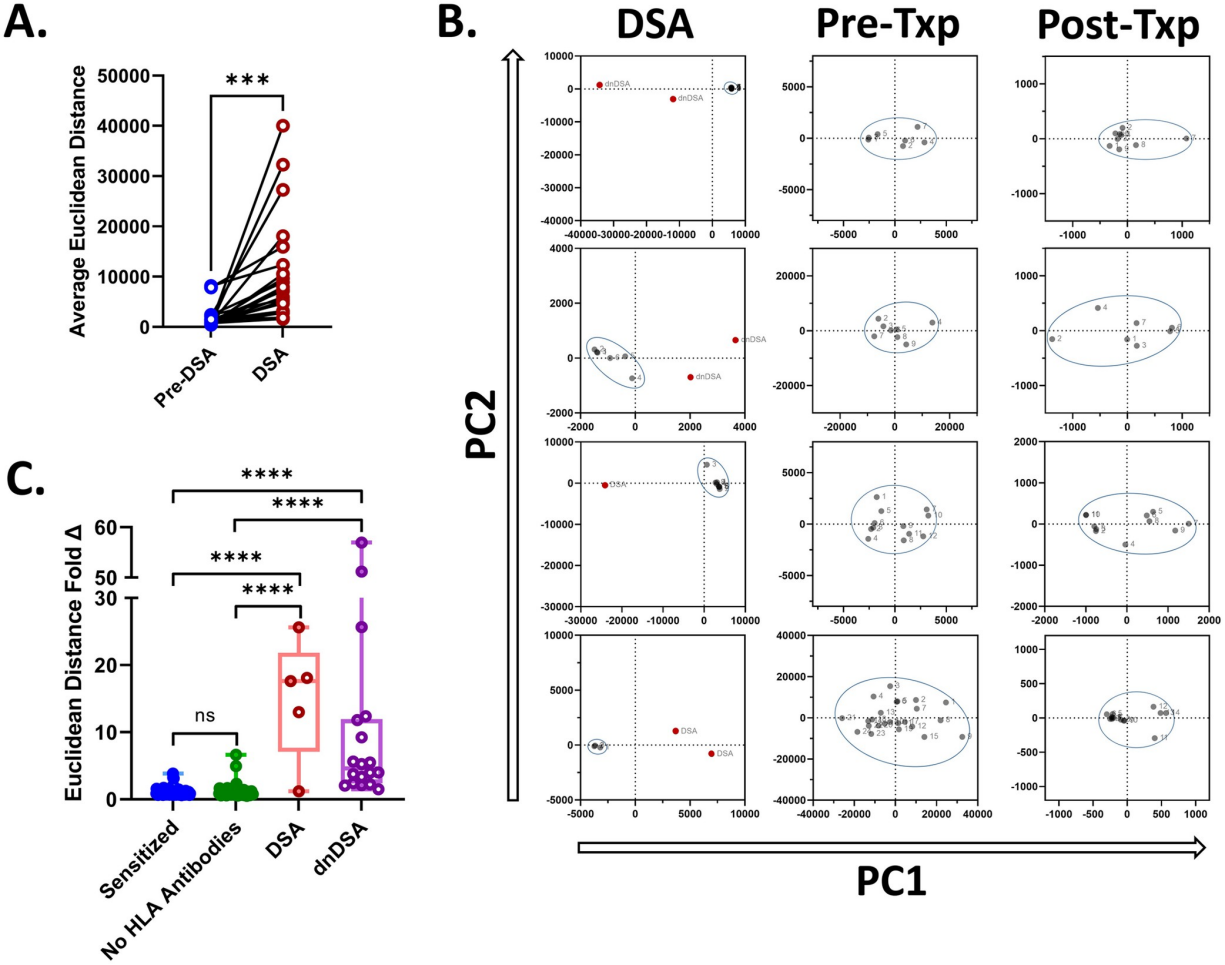
Principal component analysis of HLA class II results demonstrate that samples with class II DSAs have higher Euclidean distances. (A) For patients with class II DSAs, the average Euclidean distance observed was higher in DSA samples compared to pre-DSA samples. (B) Representative PCA dot plots for PC1 and PC2 are displayed for DSA, highly sensitized pre-transplant and low reactive post-transplant sample cases. (C) The fold difference observed between the average Euclidean distance of each sample type and the overall average Euclidean distance between patient samples was compared. ***p-value < 0.005, ****p-value <0.0001.

A similar process was followed for HLA class II samples. We found that the Euclidean distance was significantly higher for the DSA samples when they were compared to the historic pre-DSA sample Euclidean distances for our patient cohort for HLA class II (Fig 2A, paired t-test, p < 0.0005). Examples of how the PCA analysis visually appears for several patients with DSA and the two control cohorts are displayed for HLA class II (Fig 2B). For HLA class II the average Euclidean distance observed for each patient was calculated as described for the HLA class I patient samples (Fig 1C).

We observed that the Euclidean distance differences between samples post-PCA, expressed as fold change, was similar for samples that did not have drastically different changes in HLA class I (Mann-Whitney U, p = 0.0849) and HLA class II (Mann-Whitney U, p = 0.920) antibody profiles (Figs 1C and 2C, respectively). The fold change was calculated as the distance of the current sample over the average distance from all the other samples from the same patient, thus providing a ratio of how much more distance the sample being assessed exhibited in comparison to all other samples from the same patient that were considered. However, samples that had dnDSA that were identified in post-transplant testing, were more distant upon comparison using PCA, as expressed as a fold difference, in single antigen bead testing for both HLA class I (Mann-Whitney U, p < 0.0001) and HLA class II (Mann-Whitney U, p < 0.0001). The same was found for both HLA class I (Mann-Whitney U, p < 0.0001) and HLA class II (Mann-Whitney U, p < 0.0001) samples where the DSA identified was historic and not dnDSA.

**Fig 3:**
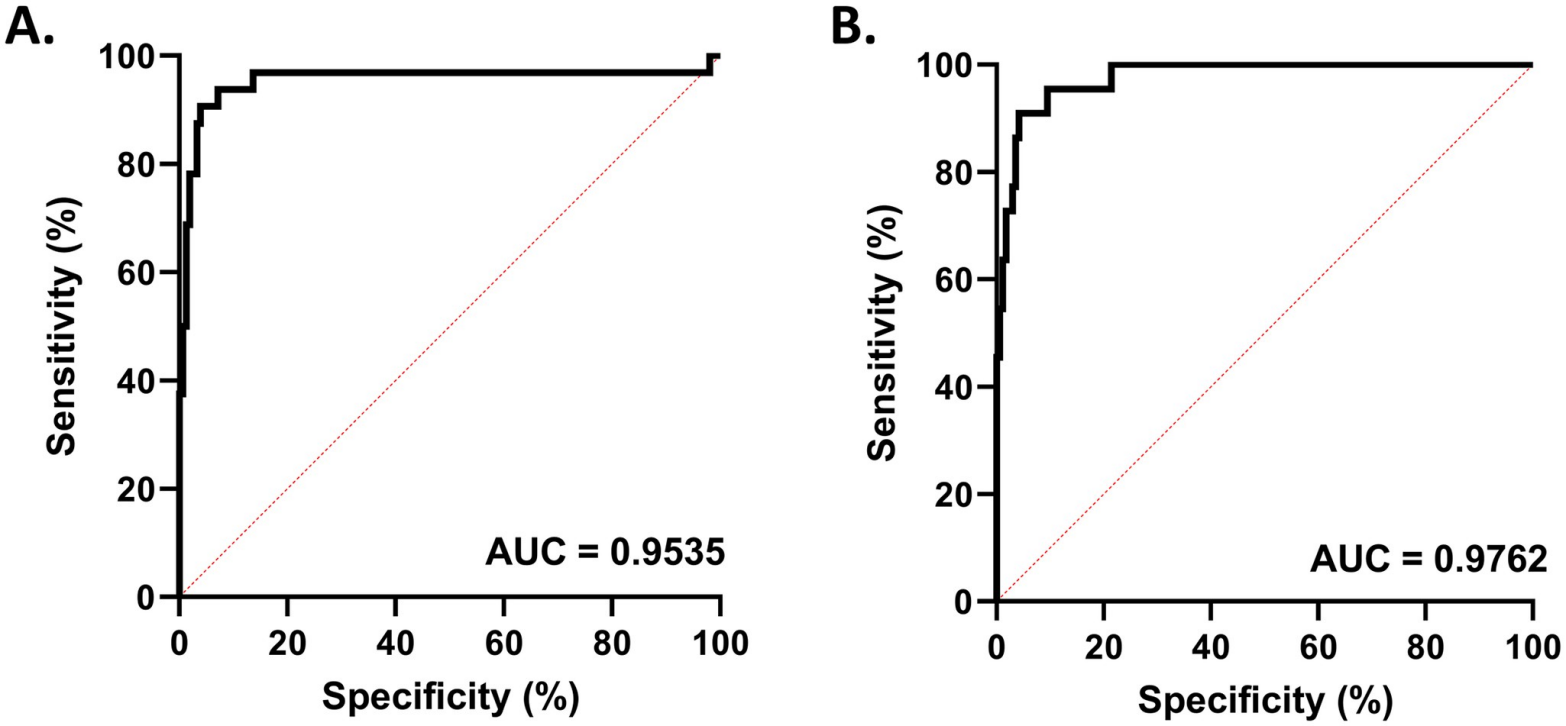
Fold differences in Euclidean distance values may differentiate samples with HLA antibody pattern deviations from patient history. (A) Receiver operator characteristic analysis performed for HLA class I single antigen bead results, demonstrating an area under the curve of 0.9535. (B) Receiver operator characteristic analysis performed for HLA class I single antigen bead results, demonstrating an area under the curve of 0.9762.

Given these findings, it seemed likely that a threshold could be established for the fold difference in Euclidean distance that could distinguish a clinically significant change in patient antibody results from PCA analysis from those that exhibited similar antibody patterns with no clinically significant change. To establish this cut-off value for both HLA class I and HLA class II antibody testing, ROC analysis was performed using the pre-transplant and post-transplant patient cohort Euclidean distance ratios as our negative controls and the Euclidean distance ratios from our patient samples with dnDSAs and DSAs as our positive control samples (Fig 3). The area under the curve (AUC) was calculated for HLA class I (Fig 3A, AUC = 0.954) and HLA class II (Fig 3B, AUC = 0.976) samples. ROC analysis found that for both HLA class I and HLA class II single antigen tests, a Euclidean distance ratio of 1.4 was optimal to distinguish the samples with DSA from the samples which have similar patterns of expression and were not clinically impactful.

To evaluate if this threshold value for HLA class I and HLA class II could autonomously identify samples with clinically significant changes in DSA antibody testing, pilot samples were tested using blinded PCA analysis on newly received samples for DSA antibody testing by the HLA class I and HLA class II single antigen bead assay. One HLA class I and one HLA class II batch were selected for the pilot study, totaling 24 HLA class I and 25 HLA class II samples respectively. After antibody testing was performed, the normalized MFI values were exported from the Fusion software before analysis (or manual review) and analyzed using the established PCA method. Samples lacking reactivity, all demonstrating bead MFI values below the assay cut-off (MFI < 1000) for positivity, were discarded from the pilot study, resulting in a total of 17 discarded samples. In total, 16 HLA class I samples and 16 HLA class II samples were included from the single antigen bead runs. Utilizing the derived principal component scores for the samples, the Euclidean distance ratio was calculated. If the Euclidean distance ratio was equal to or above 1.4, it was flagged as worthy of clinical investigation and likely abnormal from historic patient sample results. If the value was less than 1.4, the sample was not flagged and considered normal according to previous sample reactivity. These PCA results were then blindly compared to the sample’s clinically reported results which receive two manual reviews by laboratory staff and a final review by the laboratory director. The utility of PCA in screening samples for atypical HLA antibody patterns is displayed in Table 1.

**Table 1:** Performance of PCA derived anlysis for SAB Class I & II.

(N = 35)	Value (%)	95% CI
Sensitivity	100.00	73.54% to 100.00%
Specificity	78.26	56.30% to 92.54%
PPV	70.59	52.50% to 83.90%
NPV	100.00	N/A
Accuracy	85.71	69.74% to 95.19%

PCA analysis on these samples demonstrated excellent sensitivity (100%, 95% CI = 73.54% - 100%) in identifying samples that were truly abnormal and dissimilar to patient historic antibody testing (negative predictive value, 100%). Of note, PCA analysis identified all of the samples which were positive for DSA but were not *de novo* (4/4), one sample which had *de novo* DSA (1/1), and all of the samples which had irregular reactivity (7/7) compared to previous patient HLA antibody history as noted by the clinical HLA laboratory staff members requiring repeat testing, additional treatment or investigation, including one sample that exhibited complement dependent ‘prozone’ activity. The specificity for this methodology was 78.26% (95% CI = 56.30% - 92.54%) and was likely due to multiple samples for both HLA class I and HLA class II that were flagged as abnormal although they did not have DSAs and were not considered clinically different from the patients previously tested historic samples. These samples were investigated and their details are provided in Table 2. While all of these samples lacked new reactivity, they did differ from previous samples because of an increase in MFI values in one or more of the HLA specificities. The baseline MFI values for each of the specificities from these samples exhibited an increase of 2-fold or more compared to the average baseline MFI values from previous historic tests.

**Table 2:** False positive sample reactivity.

Sample ID	SAB Assay	Differnce from Historic Samples	Count of Specificities	MFI Change (%)
P002	Class I	Increased Reactivity	1	188.48%
P003	Class I	Increased Reactivity	1	169.25%
P012	Class I	Increased Reactivity	2	119.67%
P025	Class II	Increased Reactivity	2	170.80%
P033	Class II	Increased Reactivity	2	243.33%

As PCA is a method of identifying variance across multiple variables, we investigated the utility of PCA in identifying single antigen beads that might exhibit over-reactivity for the HLA class I and HLA class II solid phase assays. To investigate if PCA would appropriately identify potentially false positive beads, a cohort of patient samples was selected that had known or suspected over-reactivity for the DPA1*02:01~DPB1*01:01 and the DPA1*02:02~DPB1*05:01 beads, comprising 121 samples. These samples had baseline MFI values equal to or less than 1,200. PCA was performed using these samples with each sample utilized as a loading variable. If all beads had similar variation across samples then it would be expected that the beads would have similar PC scores and localize to a similar region of the PCA plot. The analysis revealed that while nearly all the beads exhibited similar PC scores for the two most significant eigenvectors, the DPA1*02:01~DPB1*01:01 and the DPA1*02:02~DPB1*05:01 beads exhibited a Euclidean distance that was 6.2 and 4.7 fold, respectively, more distant on average from all the other HLA class II beads (S1 Fig). Further, as expected since these samples were selected for their distinct pattern of reactivity, the DPA1*02:01~DPB1*01:01 and the DPA1*02:02~DPB1*05:01 beads aligned along similar loading vectors and localize to a similar position on the PCA plot such that, the PC values for these two beads are more similar to each other than to the other single antigen bead PC values for HLA class II.

**Fig 4:**
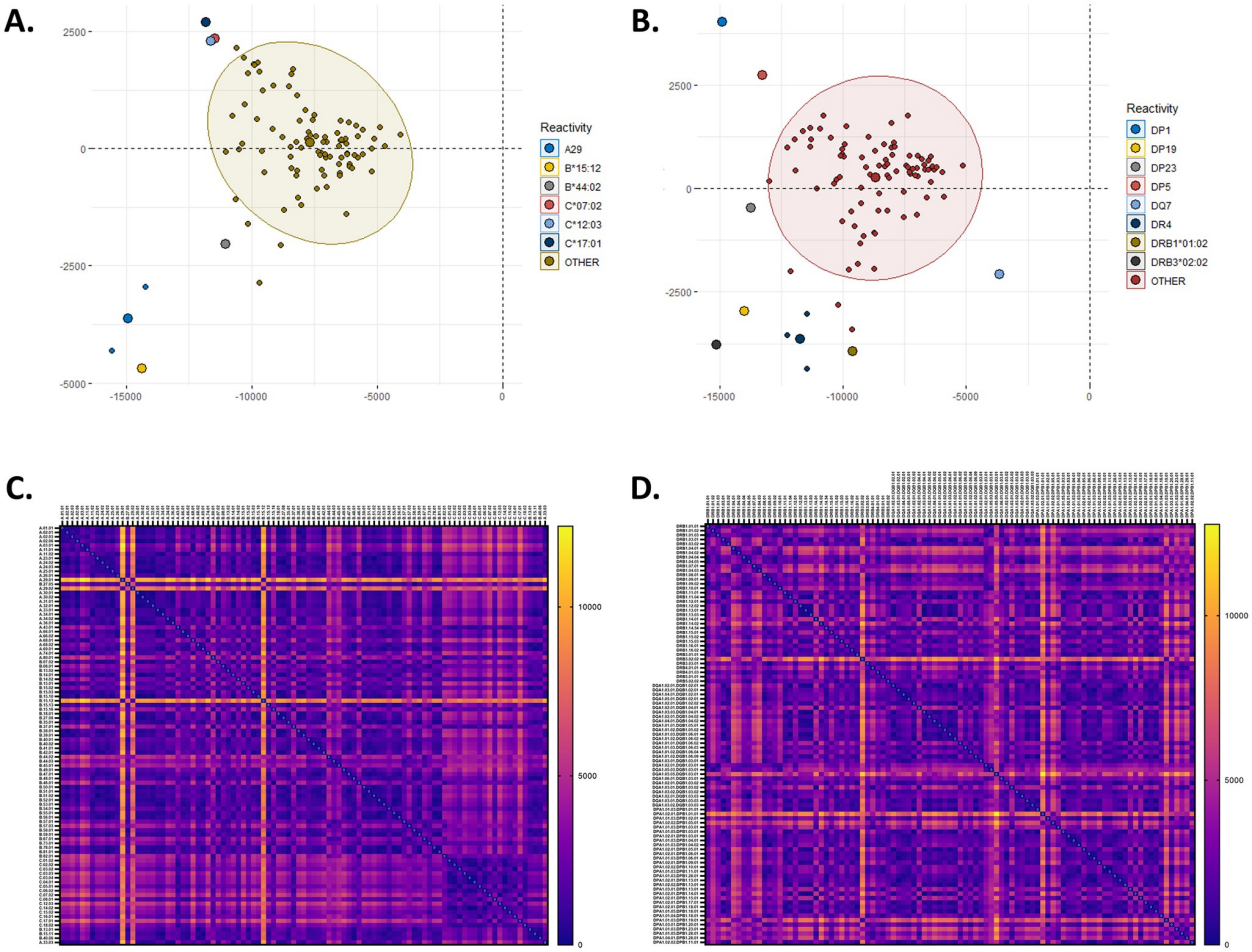
Principal component analysis of HLA class I and class II beads using a curated data set across multiple bead lots, identifies single antigen beads which display potential over reactivity. (A) Principal component analysis of class I single antigen beads, highlighting beads with the greatest Euclidean distance from all other beads. (B) Principal component analysis of class II single antigen beads, highlighting beads with the greatest Euclidean distance from all other beads. (C) Heat map demonstrating the Euclidean distance calculated post PCA for all class I beads. (D) Heat map demonstrating the Euclidean distance calculated post PCA for all class II beads.

After verifying that this method was appropriate in identifying beads with suspected over-reactivity in our pilot data set, we investigated the utility of this approach across all samples tested at the UNC HLA laboratory from 2018 through March 2022 for HLA class I (Fig 4A) and HLA class II (Fig 4B). Samples were included in the analysis if they had baseline MFI values less than 1,000. In total, there were 3,330 samples included in the analysis originally tested using the HLA class I single antigen bead assay and 3,288 samples tested using the HLA class II single antigen bead assay. Testing identified several outlying beads for HLA class I and HLA class II, representing possible overreactive beads observed commonly in our population of patients tested since 2018. For each bead, the average Euclidean distance ratio was calculated and each bead was ranked based on its distance from the average distance of all beads on the PC plot using PC1 and PC2 for both HLA class I and HLA class II (Tables 3 & 4). The HLA class I single antigen bead assay contains some beads that may be overreactive including B*15:12, C*12:03, and the A29 beads (Table 3). Several potentially overreactive beads in the HLA class II single antigen bead assay included the DRB3*02:02, DPA1*02:01-DPB1*01:01, DPA1*02.02-DPB1*05:01, DQA1*05:05-DQB1*03:01, DRB1*04:01, and DRB1*04:03 beads among others (Table 4).

**Table 3:** Class I bead PCA Euclidean distance comparison.

Rank	Specificity	Distance Ratio
1	A*29:01	3.167
2	B*15:12	2.895
3	A*29:02	2.543
4	C*17:01	1.770
5	C*12:03	1.637
6	C*07:02	1.601
7	B*44:02	1.511

**Table 4:** Class II bead PCA Euclidean distance comparison.

Rank	Specificity	Distance Ratio
1	DRB3*02:02	2.395
2	DPA1*02:01-DPB1*01:01	2.365
3	DPA1*01:03-DPB1*19:01	1.973
4	DQA1*05:05-DQB1*03:01	1.941
5	DRB1*04:01	1.759
6	DPA1*02.02-DPB1*05:01	1.740
7	DRB1*04:03	1.695
8	DPA1*01:03-DPB1*23:01	1.651

## Discussion

Multiplex solid-phase assays for the detection of HLA antibodies have transformed the field of HLA diagnostics and transplant medicine. Some of the key advantages of this method are the increased sensitivity and specificity for detecting HLA antibodies compared to cytotoxicity and other cell-based assays. As a result of these assays, there has been a sweeping change in tools that highly influence organ allocation, such as the ‘virtual crossmatch’ and ‘calculated panel reactive antibodies’ which have become essential components for center-specific immunological risk stratification for both solid and stem cell transplant, as well as kidney-paired donation programs [16–18]. It is the vital task of the transplant immunology laboratory to identify these HLA antibodies and convey the risk associated with the laboratory findings to the clinical care teams. As the task of identifying HLA antibodies becomes ever more complex, the field would greatly benefit from additional bioinformatic pipelines and software tools to assist laboratory staff in the post-analytical phase of testing [19].

As many solid organs and hematopoietic stem cell transplant patients exhibit a rather complex pattern of HLA antibodies due to various sensitizing events throughout their health care and life experiences, a computer-assisted analysis of these patterns would be ideal. Utilized for variance and pattern analysis by facial recognition software, fiscal evaluation, cyber security risk assessment, and machine learning algorithms, principal component analysis (PCA) presents a possible method for providing analysis of HLA antibody patterns, particularly those with more complex HLA antibody profiles. PCA is a dimensionality reduction technique, which uses feature extraction rather than feature exclusion, making it useful to compare variance among samples across many variables. Our study found that PCA analysis was able to enhance automated HLA antibody analysis for individual patients and assessment of bead over reactivity across a database of patient results.

Several recent manuscripts have been published by a Greece and German research group that utilized PCA to assess HLA single antigen bead data collected across three different histocompatibility centers [20, 21]. In the adult population which they investigated using principal component analysis, they found that this method could identify “patterns in human immune responses with striking similarities with the previously described CREGs” [20]. They demonstrated that anti-DP antigenic responses do not seem to be correlated to anti-DR or -DQ responses, while the latter two do tend to show a correlation based on PCA analysis [20]. In a study published a year later, they describe how through the use of PCA in their machine learning algorithm, they were able to identify several previously identified crossreactive groupings and additional new crossreactive patterns of alloreactivity [21]. A helpful feature of their study gave a detailed analysis of all bead antigenic specificities, represented as a dendrogram, calculating a measure of allelic antigenic distances for these bead-array-defined cross-reactive groups [21].

Our study utilized PCA on a more laboratory application-focused scale to preemptively analyze 24 patient HLA class I and 25 patient HLA class II HLA DSA antibody testing samples and identify samples that had deviant patterns from each patient’s historic antibody profile based on their previous testing results. Using the Euclidean distance ratio derived from principal component scores and the defined threshold which would predict a deviant antibody pattern, we compared the PCA-derived results to the laboratory staff review, testing outcomes, and finalized patient results for the HLA DSA antibody tests performed. PCA-derived analysis was able to identify all samples which had post-transplant DSA (4/4) and all other samples which had noted irregular patterns of reactivity (8/8), including one sample which exhibited a complement-mediated prozone effect. PCA had a sensitivity of 100.00% (95% CI, 73.54%-100.00%) with a negative predictive value of 100.00% across both patient cohorts.

The evaluated specificity was slightly lower at 78.26% (95% CI, 56.30%-92.54%) with a positive predictive value of 70.56% (95% CI, 52.50%-83.90%). This lower positive predictive value was due to five patient samples that PCA-derived analysis identified as deviant from historic samples, but which laboratory staff resulted with no abnormal review remarks, comments, repeat testing, or DSA and which would have been considered consistent with history by staff with no clinical impact. Each of these patient samples was investigated and compared to their historic samples. The results of these comparisons are displayed in Table 2. While there was no large visual difference when looking at the patient antibody histograms and all antibody specificities were in the same order when organized from most reactive to least reactive, there was a difference in the MFI values calculated in these five samples. In each of the five samples, the HLA specificities that historically had been above 1,000 MFIs, all exhibited at least a doubling of their MFI values, with one HLA class II sample being 243% more reactive than the average MFI values measured from the patient’s historic samples. While this increase in sample reactivity might not be clinically impactful in these five cases, cases like these must be identified and investigated as a significant increase in MFI values, as exhibited by these samples, could be clinically impactful in certain instances.

These results demonstrate evidence that PCA-assisted analysis, given the excellent negative predictive value and adequate positive predictive value, could be utilized in assisting laboratory analysis of patient samples. Depending on the laboratory workflow, this analysis could be cumbersome if it added third-party software to the existing workflow. Possibly a software package could be developed that would import the values from the.csv file export from Fusion, enabling PCA analysis of a batched run or individual samples and calculation of the Euclidean distance ratio to determine if the sample results from the run were abnormal compared to the patient’s previous history. However, if the lightweight PCA-based algorithm was added as a feature to existing software that is utilized by HLA laboratories to analyze HLA antibody data, such as HLA Fusion (OneLambda), mTilda (HLA Data Systems), HistoTrac (System Link) and/or MATCH IT (Immucor) among others, it would allow for the automated screening of samples to identify samples with significant variance from their historic antibody pattern and those which are comparable to patient antibody history. This would both simplify analysis for laboratory staff analyzing the testing results and decrease the amount of time necessary to analyze batches of patient samples, likely decreasing sample turnaround time.

An all too common complication in HLA antibody testing is the over-reactivity or false reactivity observed for some HLA single antigen beads [22, 23]. This can be the result of neo-epitope exposure and unspecific binding of serum matrix proteins. As a consequence, some single antigen beads are more prone to false reactivity or over-reactivity compared to other beads in the assays. To evaluate if PCA could provide a method for identifying this increased variance across beads that display a known pattern of over-reactivity, our study analyzed 121 single antigen bead HLA class II sample results which demonstrate overreactive DPA1*02:01-DPB1*01:01 and DPA1*02:02-DPB1*05:01 beads. Results showed that this method could distinguish the two overreactive beads from all of the other beads in the HLA class II single antigen bead panel (S1 Fig). This same method was applied to all single antigen bead HLA class I and HLA class II sample results that had been tested in our laboratory from January 2018 to March 2022 that met the sample inclusion criteria. This was done to investigate if there were beads that consistently exhibit over reactivity across multiple lots.

Our study found that for HLA class I, the beads which exhibited the most over-reactivity included A*29:01, A*29:02, and B*15:12 among others (Table 3). While the most overreactive beads for HLA class II included DRB3*02:02, DPA1*02:01-DPB1*01:01, DPA1*02.02-DPB1*05:01, DQA1*05:05-DQB1*03:01, DRB1*04:01, and DRB1*04:03 beads among other beads (Table 4). These results demonstrate that these beads have a higher variance across the thousands of patient samples tested compared to all of the other beads for the HLA class I and HLA class II single antigen bead assays. This type of analysis is beneficial for the clinical evaluation of patient results. Using this method allows the laboratory to understand which beads have the propensity to demonstrate false reactivity. Once identified, these beads merit closer scrutiny when they may have an impact on patient care.

This method can be applied to each laboratory antibody testing database once antibody MFI values have been exported from the analysis software provided by the vendor. This would provide a customized assessment of what beads display over-reactivity given the laboratory patient population and testing protocol, as certain procedures can reduce bead over-reactivity by using additional washes or treatments. This method could also be utilized to assess bead lots. If there are historic beads with a new recombinant or native HLA protein, HLA complex attachment to the beads using a different chemistry or any other production-related procedure change that could potentially impact bead reactivity upon testing, this method could be utilized to assess the bead lot(s) across one or multiple laboratory test sites.

There is a potential limitation to our study. In identifying differences in patients’ current HLA antibody patterns from historic HLA antibody patterns, we only thoroughly investigated reactivity above 1,000 MFI, since that is the cut-off for reactivity established for the assay and part of our inclusion criteria. However, it is well known that real HLA antibodies can be present and represented in these assays with MFI values below 1,000 MFI due to the spreading of antibodies over multiple beads or as a result of low concentrations in patient serum. The efficacy of this method on patient samples with only low MFI values, below 1,00 MFI, would need to be investigated with further rigor utilizing additional samples with characteristics that would best assess the performance of this method. However, it is the author’s opinion that this approach could yield similar results on samples with real HLA reactivity below 1,000 MFI. Another potential limitation is that our proof of concept study utilized only a single HLA class I and HLA class II single antigen bead run, including only a small number of patients. If the process were built and more automated so that it could be time-saving, it would be beneficial to perform a similar study and include many more patients to more stringently assess the sensitivity and specificity of the method on a larger number of patient samples.

For the implementation of these methods in the clinical laboratory, a package specific for its utility in R or incorporation of this type of method in new or existing HLA antibody analysis software would need to be completed. This would allow for practical testing of a large number of patient samples to further characterize its utility. It would also provide valuable data to demonstrate and delineate the time saved in assessing patient samples as a screening mechanism.

In summary, our study results demonstrate the utility of using PCA-derived analysis to facilitate the assessment of Luminex solid phase HLA antibody testing results. Integrating PCA-derived analysis would enable automated screening of patient samples for HLA antibody testing, identifying samples suspected of deviant HLA antibody patterns compared to previous test results. Such deviances from historic patterns could include resurgent DSA, dnDSA, increased antibody reactivity, and prozone activity. This method could also contribute to providing evidence for, and the identification of, over and/or false reactive single antigen beads in both the HLA class I and HLA class II assays.

## Supporting information

S1 Fig Analysis of a cohort of patient samples with HLA Class II bead over reactivity. Principal component analysis of a subset of patient samples with suspected DPA1*02:01-DPB1*01:01 and DPA1*02:02-DPB1*05:01 over reactivity were analyzed. PCA derived analysis revealed that the method could distinguish the suspected overreactive beads from all of the other beads from the class II single antigen bead panel.
(TIF)
