# Supplementary figures and images for: Utilizing principal component analysis in the identification of clinically relevant changes in patient HLA single antigen bead solid phase testing patterns
Source: PLoS One. 2023 Oct 26;18(10):e0288743. doi: 10.1371/journal.pone.0288743 (PMC10602234; doi:10.1371/journal.pone.0288743)

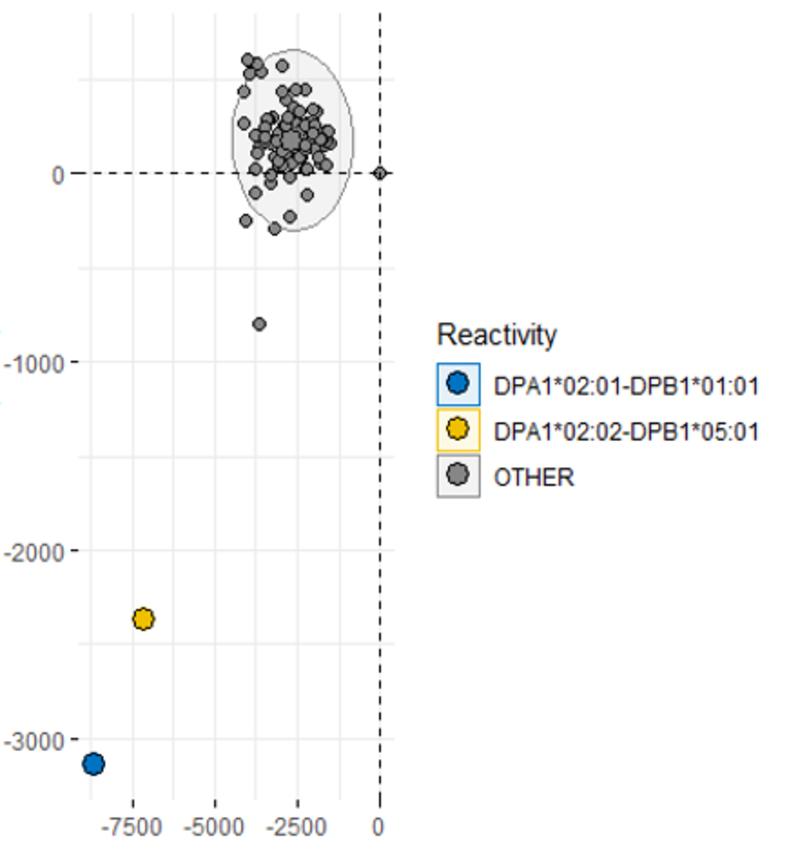

Supplement: S1 Fig — Principal component analysis of a subset of patient samples with suspected DPA1*02:01-DPB1*01:01 and DPA1*02:02-DPB1*05:01 over reactivity were analyzed. PCA derived analysis revealed that the method could distinguish the suspected overreactive beads from all of the other beads from the class II single antigen bead panel. (TIF) [file pone.0288743.s001.tif]
